# Addition of tert-butylhydroquinone (TBHQ) to maize oil reduces lipid oxidation but does not prevent reductions in serum vitamin E in nursery pigs

**DOI:** 10.1186/s40104-019-0362-5

**Published:** 2019-07-04

**Authors:** Yuan T. Hung, Andrea R. Hanson, Pedro E. Urriola, Lee J. Johnston, Brian J. Kerr, Gerald C. Shurson

**Affiliations:** 1 Department of Animal Science, University of Minnesota, 1988 Fitch Ave., St. Paul, MN 55108 USA; 2 Professional Swine Management, LLC, Carthage, IL 62321 USA; 3 USDA-ARS National Laboratory for Agriculture and the Environment, Ames, IA 50011 USA; 4 West Central Research and Outreach Center, University of Minnesota, Morris, MN 56267 USA

**Keywords:** Growth performance, Lipid oxidation, Maize oil, Nursery pigs, TBHQ, Vitamin E

## Abstract

**Background:**

Maize oil is abundantly used in foods and feeds and is highly susceptible to oxidation. Consequently, commercially available antioxidants should be evaluated for effectiveness against lipid oxidation in swine diets. Our study was conducted to evaluate growth performance of nursery pigs fed oxidized maize oil and to determine effects of using antioxidants on oxidative status in a 2 × 2 factorial design. Two hundred eight weaned pigs were blocked by initial BW into 13 blocks, resulting in 4 pigs per pen and 13 pens per treatment. Dietary treatments included 6% unoxidized or oxidized maize oil, and 0 or 60 mg/kg of tert-butylhydroquinone (TBHQ), which was added after lipid oxidation. Data for growth performance were collected from 5 time periods of a two-phase feeding program (Phase 1 = d 0 to 12 and Phase 2 = d 13 to 34). Serum and liver samples were collected from one pig per pen, which had initial BW closest to average BW to determine oxidative status on d 34.

**Results:**

Oxidized maize oil was heated for 12 h at 185 °C with 12 L/min of air, yielding a peroxide value (PV) of 5.98 mEq O_2_/kg and TBARS of 0.11 mg MDA eq/g. Addition of TBHQ to diets containing oxidized maize oil decreased PV by 37% and increased the oil stability index by 69%. Final BW, ADG, ADFI, and G:F of pigs were not different among the four dietary treatments. However, pigs fed oxidized maize oil tended (*P* <  0.08) to increase hepatosomatic index by 5% compared with those fed unoxidized oil, and this was not affected by adding TBHQ. The serum vitamin E concentration of pigs fed oxidized maize oil was less (*P <* 0.03) than pigs fed unoxidized oil, but this reduction was not reversed by adding TBHQ. Finally, the serum and liver selenium concentration were not different among the treatments.

**Conclusions:**

The addition of TBHQ did not affect growth performance and vitamin E status in pigs fed moderately oxidized maize oil, but TBHQ reduced lipid oxidation, enhanced the oil stability, and appeared to reduce oxidative stress.

## Background

Maize oil is an abundant source of vegetable oil used in human foods and animal feeds. Maize oil provides 8579 kcal/kg of ME [1], which is comparable to soybean oil (8574 kcal/kg), and greater than canola oil (8384 kcal/kg) and palm kernel oil (7119 kcal/kg) when added to swine diets. The addition of lipids to animal feeds not only increases energy density, but also enhances the absorption of fat-soluble vitamins and improves feed efficiency and palatability [2]. The U.S. ethanol industry produces about 1.84 billion kg of distillers maize oil annually, which is used in swine and poultry feeds as well as biodiesel feedstock [3]. However, recent studies have reported that distillers maize oil sources vary in the extent of oxidation [4, 5]. Because maize oil contains high concentrations of unsaturated fatty acids, which can range from 81% to 95% of total fatty acids [4, 6], it is highly susceptible to oxidation when storage and processing conditions involve exposure to oxygen, heat, and pro-oxidant metals [7].

Several negative effects of feeding oxidized lipids to poultry and swine have been summarized [8], including reduced energy digestibility [9–11], growth rate [11, 12], feed intake [13], feed efficiency [14, 15], and impaired immune function and oxidative status [14, 16]. Furthermore, some studies observed that feeding diets containing oxidized lipids increased the number of pigs medicated [15] and mortality rate in broilers [17]. Therefore, there is a need to find approaches to prevent lipid oxidation of fats and oils used in animal feeds to minimize these negative effects on animal health and growth performance.

Although maize oil contains significant amounts of natural antioxidant compounds (tocopherols, tocotrienols, phytosterols, steryl ferulates, and carotenoids) [18], synthetic antioxidants are considered to be more effective and stable during processing [19]. Several commonly used commercial antioxidant products include butylated hydroxyanisole (BHA), butylated hydroxytoluene (BHT), ethoxyquin, tert-butylhydroquinone (TBHQ), and propyl gallate. Compared to BHA and BHT, TBHQ has superior protection in vegetable oil due to its stability at high temperatures [20, 21]. In addition, a synergistic effect has been observed for TBHQ when used in combination and other antioxidants (e.g. citrate, BHA, and BHT) to increase the thermal stability and smoke point of fats and oils [21, 22]. The addition of TBHQ and a blend of ethoxyquin and TBHQ have been shown to significantly reduce lipid oxidation of distillers’ maize oil when stored under hot (38.6 °C) and humid (94% relative humidity) conditions for 28 d [23]. Nevertheless, the potential benefits of feeding lipids containing synthetic antioxidants have not been extensively evaluated, and responses have been inconsistent. Dietary antioxidants tended to improve G:F in finishing barrows during the first 28 d, but not for the entire feeding period [16]. Therefore, we hypothesized that the addition of TBHQ to maize oil supplemented in nursery pig diets would prevent further lipid oxidation and ameliorate the potential negative effects on growth performance and oxidative stress. The objective of this study was to investigate the effects of adding TBHQ to unoxidized and oxidized maize oil on growth performance and oxidative status of nursery pigs.

## Materials and methods

The University of Minnesota Institutional Animal Care and Use Committee approved the experimental design and animal use under protocol 1304-30545A.

### Animals and experimental design

This experiment was conducted at the University of Minnesota West Central Research and Outreach Center in Morris, MN. Crossbred barrows and gilts (*n* = 208) were weaned at 21 days of age (initial BW = 7.6 ± 0.6 kg) and fed experimental diets during a 34-d feeding period, using a two-phase feeding program (Phase 1 = d 0 to 12 and Phase 2 = d 13 to 34). Pigs were housed in 2 identical nursery rooms and stratified by BW into 13 blocks. Pens of pigs (4 pigs/pen) within block were assigned randomly to 1 of 4 treatments in a 2 × 2 factorial arrangement, resulting in 13 pens per treatment. Each pen (2.4 m × 1.2 m) consisted of plastic grated flooring with a dry feeder (4 feeder spaces) and a nipple drinker with cup.

### Maize oil treatments

Refined, deodorized, bleached maize oil (Stratas Foods, LLC, Memphis, TN) was purchased and used in this study. This maize oil source had an initial peroxide value (PV) of 0.05 mEq O_2_/kg, and 0.04% free fatty acids. Half of the amount of fresh maize oil was subjected to oxidation by heating it at 185 °C for 12 h with 12 L/min continuous flow of compressed air. The other half of fresh maize oil was not heated (unoxidized). After heating exposure, both oxidized and unoxidized maize oil samples were stored in barrels at − 20 °C to prevent further oxidation. At 7 d prior to weaning and the start of the experiment, half of the oil samples from oxidized and unoxidized oil were mixed with Rendox® CQ (Kemin Industries, Des Moines, IA) to supply 60 mg/kg of TBHQ prior to mixing experimental diets. Hence, the TBHQ was added after the heating process. The oxidized and unoxidized maize oil batches, with and without TBHQ, were moved to the feed mill and added at 6% to the phase 1 diets (Table 1). After mixing the phase 1 diets, maize oil samples were stored in the feed mill for 15 d until mixing the phase 2 diets.

**Table 1:** Ingredient and nutrient composition of experimental diets (as-fed basis)

Phase	Phase 1 (Weaning – d 12)				Phase 2 (d 13 – d 34)			
Maize oil	Unoxidized		Oxidized		Unoxidized		Oxidized	
Antioxidant^a^	–	+	–	+	–	+	–	+
Ingredient composition, %								
Maize	36.67	36.67	36.67	36.67	55.34	55.34	55.34	55.34
Soybean meal, 47.5% CP	18.01	18.01	18.01	18.01	34.04	34.04	34.04	34.04
Unoxidized maize oil^b^	6.00	.	.	.	6.00	.	.	.
Unoxidized maize oil + TBHQ	.	6.00	.	.	.	6.00	.	.
Oxidized maize oil^c^	.	.	6.00	.	.	.	6.00	.
Oxidized maize oil + TBHQ	.	.	.	6.00	.	.	.	6.00
Dried whey	24.31	24.31	24.31	24.31	.	.	.	.
Soy protein isolate	10.00	10.00	10.00	10.00	.	.	.	.
Zinc oxide	0.50	0.50	0.50	0.50	.	.	.	.
Antibiotics^d^	0.57	0.57	0.57	0.57	.	.	.	.
Vitamin/mineral premix^e^	0.50	0.50	0.50	0.50	0.50	0.50	0.50	0.50
*L*-Lys	0.43	0.43	0.43	0.43	0.53	0.53	0.53	0.53
*DL*-Met	0.26	0.26	0.26	0.26	0.21	0.21	0.21	0.21
*L*-Thr	0.12	0.12	0.12	0.12	0.17	0.17	0.17	0.17
Monocalcium phosphate	1.03	1.03	1.03	1.03	1.16	1.16	1.16	1.16
Limestone	1.25	1.25	1.25	1.25	1.28	1.28	1.28	1.28
Salt	0.35	0.35	0.35	0.35	0.77	0.77	0.77	0.77
Total	100.0	100.0	100.0	100.0	100.0	100.0	100.0	100.0
Calculated composition								
ME^f^, kcal/kg	3619	3619	3619	3619	3556	3556	3556	3556
Crude protein, %	23.88	23.88	23.88	23.88	21.56	21.56	21.56	21.56
NDF, %	4.82	4.82	4.82	4.82	7.83	7.83	7.83	7.83
Ether extract, %	8.07	8.07	8.07	8.07	8.44	8.44	8.44	8.44
Linoleic acid, %	3.88	3.88	3.88	3.88	4.26	4.26	4.26	4.26
ATTD^g^ P, %	0.39	0.39	0.39	0.39	0.33	0.33	0.33	0.33
Ca, %	0.90	0.90	0.90	0.90	0.81	0.81	0.81	0.81
SID^h^ Lys, %	1.56	1.56	1.56	1.56	1.42	1.42	1.42	1.42
SID Met/Cys, %	0.86	0.86	0.86	0.86	0.78	0.78	0.78	0.78
SID Thr, %	0.91	0.91	0.91	0.91	0.83	0.83	0.83	0.83
SID Trp, %	0.26	0.26	0.26	0.26	0.23	0.23	0.23	0.23
Lactose, %	17.50	17.50	17.50	17.50	0.00	0.00	0.00	0.00
Vitamin E, IU/kg	22.00	22.00	22.00	22.00	22.00	22.00	22.00	22.00
g SID Lys:Mcal ME	4.31	4.31	4.31	4.31	3.99	3.99	3.99	3.99
g ATTD P:Mcal ME	1.08	1.08	1.08	1.08	0.93	0.93	0.93	0.93
Ca:ATTD P	2.31	2.31	2.31	2.31	2.45	2.45	2.45	2.45
SID Met + Cys:SID Lys	55.13	55.13	55.13	55.13	54.93	54.93	54.93	54.93
SID Thr:SID Lys	58.33	58.33	58.33	58.33	58.45	58.45	58.45	58.45
SID Trp:SID Lys	16.67	16.67	16.67	16.67	16.20	16.20	16.20	16.20
Analyzed composition, %								
Dry matter	91.14	91.33	91.13	91.17	88.39	88.18	88.21	88.10
Ether extract	4.72	5.01	4.49	3.81	4.12	4.22	3.79	3.77
Crude fiber	2.15	2.04	2.04	1.88	3.02	3.26	2.94	2.73
Ash	7.38	7.20	7.06	7.05	5.46	5.01	6.02	6.01
Crude protein	22.89	23.72	23.98	23.50	21.28	21.22	22.78	21.60
Lysine	1.62	1.80	1.72	1.83	1.52	1.54	1.59	1.67
Methionine	0.50	0.50	0.54	0.51	0.46	0.41	0.49	0.45
Cysteine	0.32	0.33	0.32	0.33	0.31	0.31	0.34	0.34
Threonine	0.99	1.05	1.03	1.06	0.93	0.94	0.99	1.01
Tryptophan	0.32	0.32	0.33	0.35	0.29	0.28	0.30	0.27

^a^Antioxidant, Rendox CQ (active ingredient is TBHQ; Kemin Industries, Des Moines, IA) was added at 1000 mg/kg in maize oil

^b^Stratas Foods, LLC

^c^Maize oil heated for 12 h at 185 °C with a constant air flow rate of 12 L/min

^d^Antibiotics added were 0.175% Denagard (tiamulin 22 g/kg; Boehringer Ingelheim Vetmedica, Inc., St. Joseph, MO) and 0.4% Aureomycin 50G (chlortetracycline 110 mg/kg; Zoetis, Inc., Florham Park, NJ), which provided 38.5 mg tiamulin per kg of diet, and 440 mg chlortetracycline per kg of diet

^e^Premix for nursery pigs (< 22.5 kg) at 0.5% dietary inclusion rate provided the following nutrients per kilogram of feed: 11023 IU of vitamin A as retinyl acetate; 2756 IU of vitamin D_3_; 22 IU of vitamin E as *DL*-alpha tocopheryl acetate; 4.41 mg of vitamin K as menadione dimethylpyrimidinol bisulfite; 9.92 mg of riboflavin; 55.11 mg of niacin; 33.07 mg of pantothenic acid as *D*-calcium pantothenate; 992 mg of choline as choline chloride; 0.06 mg of vitamin B_12_; 14.3 mg of pyridoxine; 1.65 mg of folic acid; 2.20 mg of thiamine; 0.33 mg of biotin; 2.20 mg of iodine as ethylenediamine dihydroiodide; 0.30 mg of selenium as sodium selenite; 299 mg of zinc as zinc sulfate; 299 mg of iron as ferrous sulfate; 19.8 mg of copper as copper sulfate; and 17.6 mg of manganese as manganese oxide

^f^ME = metabolizable energy

^g^ATTD = apparent total tract digestible

^h^SID = standardized ileal digestible

Maize oil samples were retained during manufacture of phase 1 and 2 diets at − 20 °C until the end of the growth performance study, and were subsequently analyzed for oxidation at the University of Missouri Agricultural Experiment Station Chemistry Laboratory using standard AOCS procedures [24] for PV (method Cd 8–53), thiobarbituric acid reactive substances (TBARS), p-anisidine value (AnV), and oil stability index (OSI). The TBARS value was modified from AOCS procedure (method Cd 19–90), which uses malonaldehyde (MDA) as a standard as described by Pegg [25]. The OSI at 110 °C was determined by the AOCS official method (method Cd 12b − 92) [24]. The AnV is a relative measurement to determine the concentration of aldehydes in lipid after hydroperoxides were decomposed, and was determined using method Cd 18–90 at the Agricultural Experimental Station Analytical Chemistry Laboratory of the University of Missouri.

### Experimental diets

Diets were isocaloric and consisted of maize and soybean meal with 6% unoxidized or oxidized maize oil in combination with 0 or 60 mg/kg of TBHQ (Rendox® CQ). Phase 1 diets contained dried whey and soy protein isolate to minimize the inclusion and antigenic effects of soybean meal. Zinc oxide (3600 mg/kg) and antibiotics (tiamulin - 38.5 mg/kg and chlortetracycline - 440 mg/kg diet) were also included in Phase 1 diets to minimize post-weaning health concerns, such as enteric diseases and diarrhea. All diets were formulated to exceed the nutritional requirements of weaned pigs suggested by NRC (2012) [1], with an additional safety margin of 5% for lysine, methionine + cysteine, tryptophan, and vitamin E. All pigs were provided *ad libitum* access to their assigned experimental diets, which were fed in meal form in both nursery phases.

Feed samples were retained, frozen at − 20 °C, and analyzed by following AOAC official method [26] for dry matter (method 930.15), crude fat (method 920.39), crude fiber (method 978.10), crude protein (method 990.03), and ash (method 942.05) at Minnesota Valley Testing Laboratories (New Ulm). Lysine, methionine, cysteine, threonine, and tryptophan were analyzed (method 982.30) at the University of Missouri Agricultural Experiment Station Chemistry Laboratory (Columbia).

### Data and sample collection

Individual pigs and feeders in each pen were weighed on days 0, 5, 12, 19, 26, and 34 (end of trial) post-weaning to calculate pen average daily gain (ADG), average daily feed intake (ADFI), and gain to feed ratio (G:F). For blood and tissue collection, focal pigs (13 pigs/treatment) were selected based on the individual pig closest to the mean initial BW in each pen. Blood (10 mL) was collected via jugular venipuncture using trace element-free serum tubes (Cat #368380; Becton Dickinson, Franklin Lakes, NJ) on d 34 (fed state). Blood samples were placed on ice for clotting after collection and centrifuged at 1400×*g* for 10 min at 4 °C. Serum was transferred into microcentrifuge tubes and frozen at − 20 °C for subsequent selenium (Se) and vitamin E analysis. At the end of trial (d 34), all focal pigs were euthanized by captive bolt followed by exsanguination. Intact livers were excised, weighed individually, and the hepatosomatic index (HSI) was calculated as wet liver weight (kg)/body weight (kg). Subsequently, portions of the liver were harvested and stored at − 80 °C until analysis of Se and vitamin E concentration.

### Selenium and vitamin E analysis in serum and liver

The Se and vitamin E concentrations in serum and liver were analyzed at the Michigan State University Diagnostic Center for Population and Animal Health (East Lansing). One gram of liver tissue was digested overnight in 2 mL nitric acid, and Se concentrations were determined according to the procedure of Wahlen et al. [27] of using an inductively coupled plasma mass spectrometer (Agilent 7500ce, Agilent Technologies, Inc., Santa Clara, CA). For vitamin E analysis, liver samples were weighed and homogenized in distilled, deionized water (1:4 *w*/*v*). Serum samples and liver homogenates were mixed with equal volumes of hexane and a solution of BHT in ethanol (10% *w*/*v*). Mixtures were centrifuged at 1900×*g* for 10 min, and a known aliquot of the hexane layer was removed and dried under vacuum. Samples were dissolved in a chromatographic mobile phase (7:2:1, acetonitrile, methylene chloride, methanol) and analyzed by HPLC (Separation Module 2690) using an analytical column (Waters Symmetry C18, 3.5 mm, 4.6 μm × 75 mm) with detection by UV absorbance at 292 nm (Waters, Milford, MA). Trans-β-APO-8″-carotenal was used as an internal standard.

### Statistical analysis

Data were analyzed for overall structure, absence of outliers, and normal distribution using the PROC UNIVARIATE procedure of SAS (SAS Institute, Cary, NC). Experimental data were analyzed as a randomized complete block design using PROC MIXED of SAS. Pen was considered as the experimental unit for all responses. The statistical model included fixed effects of extent of maize oil oxidation, time, antioxidant, as well as 2- and 3-way interactions. Random effects included block and pen. Data were analyzed for the effect of time by REPEATED measures, and an unstructured covariance matrix was used. Data for liver weight, HSI, as well as hepatic and serum vitamin E and Se were analyzed with a similar treatment structure, but the effect of time was not included in the model. Interactions were removed from the model when there was no significant effect. All results were reported as least squares means. Multiple comparisons among treatments were performed using PDIFF and adjusted by the Tukey option for multiple comparisons of means. Significant differences were declared at *P <* 0.05 and statistical trends at *P <* 0.10.

## Results

### General observations

Due to poor health, 8 pigs either died or were removed from 3 of the 4 experimental treatments (3 pigs from unoxidized oil without TBHQ, 3 pigs from unoxidized oil with TBHQ, and 2 pigs from oxidized oil without TBHQ), and none died or were removed for the treatment of oxidized oil with TBHQ, so these pigs were not included in the data set. No outliers were identified and no data required removal. However, there were signs of acute diarrhea and growth depression between d 5 and d 12 post-weaning. Therefore, based on the attending veterinarian’s recommendation, all pigs were treated with neomycin sulfate (22 mg/kg BW) for 3 d, then tiamulin hydrogen fumarate (23 mg/kg BW) for 5 d by water medication to reduce the impact of diarrhea on the growth performance responses. However, after administering the water medication, suboptimal health continued for this group of pigs, which affected the growth performance responses observed in the study.

### Oxidation analysis of maize oil

Maize oil samples were analyzed for concentrations of PV, TBARS, AnV, and OSI. The PV of fresh, unoxidized maize oil was 1.85 mEq O_2_/kg, which was greater than the manufacturer specification (0.05 mEq O_2_/kg; Table 2). Maize oil heated at 185 °C for 12 h with air resulted in greater concentrations of PV, TBARS, and AnV than unheated maize oil, which indicated that the heating process and temperature used achieved oxidation. For the maize oil used in phase 1 diets, the PV of the oxidized oil with TBHQ added after heating was 63% less than the oxidized maize oil without TBHQ. However, TBARS and AnV concentrations were not affected by addition of TBHQ to both oxidized and unoxidized maize oil. Oil stability index was 20.85 h in the unoxidized maize oil and 16.05 h in the oxidized maize oil. When TBHQ was added to unoxidized and oxidized maize oil, the OSI increased by 36% and 69%, respectively.

**Table 2:** Lipid oxidation indices of maize oil used in the experiment

Maize oil^a^	Unoxidized		Oxidized	
Antioxidant^b^	–	+	–	+
Oil used in phase 1 diets				
Peroxide value, mEq O_2_/kg^c^	1.85	1.99	5.98	3.75
TBARS, mg MDA/g^d,e^	0.06	0.06	0.11	0.12
AnV^f^	3.0	3.1	134.9	131.9
Oil stability index, h	20.85	28.30	16.05	27.20
Oil used in phase 2 diets				
Peroxide value, mEq O_2_/kg	1.95	2.00	5.99	5.89
TBARS, mg MDA/g	0.06	0.06	0.11	0.11
AnV	3.1	3.3	140.8	142.0
Oil stability index, h	10.25	23.15	4.65	11.15

^a^Oils were sampled after manufacturing phase 1 and 2 diets (15 d after mixing phase 1 diets), and stored at −20 °C until analysis at the University of Missouri Agricultural Experiment Station Chemistry Laboratory

^b^Antioxidant = Rendox CQ (active ingredient is TBHQ; Kemin Industries, Des Moines, IA) was added at 1000 mg/kg in maize oil

^c^Milliequivalents peroxide per kg of oil. W/W% = g/100 g of sample

^d^TBARS = thiobarbituric acid reactive substances

^e^MDA = malondialdehyde

^f^AnV = p-anisidine value

The concentration of PV, TBARS, and AnV of the maize oil used in the phase 2 diets did not change dramatically due to the addition of TBHQ. Nevertheless, adding TBHQ in both unoxidized and oxidized maize oil increased the time of OSI more than 2 times compared with no addition of TBHQ. After mixing phase 1 diets, the oil was stored in the feed mill until mixing phase 2 diets, so the OSI was expected to be reduced during storage. The OSI of unoxidized maize oil decreased from 20.85 h in phase 1 to 10.25 h in phase 2, but adding TBHQ to unoxidized maize oil resulted in the OSI decreasing by only 5 h. Furthermore, the OSI in the oxidized maize oil without TBHQ decreased by 71% (16.05 to 4.65 h,) while the OSI of the oxidized maize oil with TBHQ decreased by 59% (27.2 to 11.15 h). These results indicate that TBHQ was partially effective in stabilizing both oxidized and unoxidized maize oil based on changes in OSI.

### Effect of oxidized maize oil and TBHQ on growth performance

There were no differences in initial BW among four dietary treatments (Table 3). Feeding diets containing oxidized maize oil, with or without TBHQ addition, did not affect BW (*P* > 0.48) on d 5, 12, 19, 26, and final BW on d 34. Similarly, the ADG in any of the feeding periods was not affected by feeding diets containing oxidized maize oil, with or without the addition of TBHQ, and there was no interaction between maize oil oxidation and antioxidant (*P* > 0.44; Table 4). Furthermore, there was no effect of feeding diets containing oxidized maize oil, with or without TBHQ, on ADFI on d 5, 12, 19, 26, and overall (*P >* 0.27; Table 5). As a result of no differences in ADG and ADFI among dietary treatments, there were no effects of dietary treatments on G:F (Table 6).

**Table 3:** Effects of feeding maize oil and TBHQ on body weight of nursery pigs

Maize oil	Unoxidized		Oxidized	
Antioxidant^a^	–	+	–	+
Body weight, kg				
Initial	7.6	7.6	7.6	7.6
D 5	7.6	7.6	7.6	7.5
D 12	9.9	9.8	9.8	9.4
D 19	12.5	12.3	12.3	11.7
D 26	15.8	15.5	15.5	14.8
D 34	20.5	20.1	20.2	19.2
Pooled-SEM	0.60			
*P*-value				
Oxidation	0.53			
Antioxidant	0.48			
Oxidation × Antioxidant	0.73			
Day	< 0.01			
Day × Oxidation	0.26			
Day × Antioxidant	0.09			
Day × Oxidation × Antioxidant	0.84			

^a^Rendox® CQ, (active ingredient is TBHQ; Kemin Industries, Des Moines, IA) was added at 1000 mg/kg of lipid

**Table 4:** Effects of feeding maize oil and TBHQ on average daily gain of nursery pigs

Maize oil	Unoxidized		Oxidized	
Antioxidant^a^	–	+	–	+
Average daily gain, g				
D 0–5	16	2	1	-9
D 5–12	324	310	322	265
D 12–19	356	361	351	329
D 19–26	474	462	465	444
D 26–34	629	628	634	601
Overall (day 0–34)	360	353	355	326
Pooled-SEM	14			
*P*-value				
Oxidation	0.25			
Antioxidant	0.20			
Oxidation × Antioxidant	0.44			
Day	< 0.01			
Day × Oxidation	0.99			
Day × Antioxidant	0.84			
Day × Oxidation × Antioxidant	0.89			

^a^Rendox® CQ, (active ingredient is TBHQ; Kemin Industries, Des Moines, IA) was added at 1000 mg/kg of lipid

**Table 5:** Effects of feeding diets containing maize oil and TBHQ on feed intake of nursery pigs

Maize oil	Unoxidized		Oxidized	
Antioxidant^a^	–	+	–	+
Average daily feed intake, g				
D 0–5	99	97	90	82
D 5–12	395	369	374	311
D 12–19	704	703	711	716
D 19–26	734	689	703	683
D 26–34	999	943	964	972
Overall (day 0–34)	586	560	568	553
Pooled-SEM	19			
*P*-value				
Oxidation	0.50			
Antioxidant	0.27			
Oxidation × Antioxidant	0.79			
Day	< 0.01			
Day × Oxidation	0.83			
Day × Antioxidant	0.81			
Day × Oxidation × Antioxidant	0.82			

^a^Rendox® CQ, (active ingredient is TBHQ; Kemin Industries, Des Moines, IA) was added at 1000 mg/kg of lipid

**Table 6:** Effects of feeding diets containing maize oil and TBHQ on gain efficiency of nursery pigs

Maize oil	Unoxidized		Oxidized	
Antioxidant^a^	–	+	–	+
Gain:Feed, g/kg				
D 0–5	−38	−199	− 308	− 344
D 5–12	858	941	882	843
D 12–19	519	525	499	458
D 19–26	648	680	665	654
D 26–34	658	682	669	619
Overall (D 0–34)	529	526	481	446
Pooled-SEM	58			
*P*-value				
Oxidation	0.27			
Antioxidant	0.73			
Oxidation × Antioxidant	0.78			
Day	< 0.01			
Day × Oxidation	0.80			
Day × Antioxidant	0.97			
Day × Oxidation × Antioxidant	0.97			

^a^Rendox® CQ, (active ingredient is TBHQ; Kemin Industries, Des Moines, IA) was added at 1000 mg/kg of lipid

### Effect of oxidized maize oil and TBHQ on oxidative stress

There was no interaction between maize oil oxidation and antioxidant on liver and serum oxidative status (Table 7). Liver weight, as well as liver Se and vitamin E concentrations, were not affected by feeding oxidized maize oil with or without the addition of TBHQ. However, the HSI tended to be greater (*P* = 0.08) for pigs consuming oxidized maize oil compared with those fed unoxidized maize oil, but was not affected by adding TBHQ to maize oil. In serum, the Se concentration was not different in pigs consuming oxidized maize oil (*P* > 0.40) compared with unoxidized maize oil, with or without the addition of TBHQ (*P* > 0.32). In contrast, pigs fed oxidized maize oil had reduced (*P* = 0.03) serum vitamin E concentrations compared with those consuming the diet with unoxidized oil, but adding TBHQ to unoxidized and oxidized maize oil did not significantly increase serum vitamin E concentrations in pigs.

**Table 7:** Serum and tissue parameters of nursery pigs fed maize oil and TBHQ

Maize oil	Unoxidized		Oxidized		SEM	*P*-values		
Antioxidant^a^	–	+	–	+		OX^b^	AX^c^	OX × AX^d^
Liver								
Weight, g	583	605	633	621	33	0.32	0.88	0.60
HSI^e^	2.96	3.08	3.17	3.20	0.09	0.08	0.42	0.65
Se, μg/g dry weight	2.55	2.45	2.53	2.56	0.06	0.44	0.61	0.31
Vitamin E, μg/g dry weight^f^.	29.12	26.61	26.12	24.88	2.66	0.38	0.48	0.81
Serum								
Se, ng/mL	173	163	164	164	6	0.40	0.32	0.39
Vitamin E, μg/mL	0.43	0.54	0.38	0.38	0.05	0.03	0.23	0.26

^a^Rendox® CQ, (active ingredient is TBHQ; Kemin Industries, Des Moines, IA) was added at 1000 mg/kg of lipid

^b^OX = oxidation

^c^AX = antioxidant

^d^OX × AX = interaction effect between oxidation and antioxidant

^e^HSI = hepatosomatic index = liver weight as % of BW

^f^Vitamin E was measured in α-tocopherol equivalents

## Discussion

Peroxide value, TBARS, AnV, and OSI are common assays used to characterize lipid oxidation of fats and oils in the feed industry [28]. However, each assay measures only a fraction of various types of oxidation products, which accumulate and degrade over time [29]. Therefore, multiple oxidation measures are required for a more comprehensive assessment of the extent of oxidation of fats and oils [29]. Results from the current study showed that PV, TBARS, and AnV content was increased when maize oil was heated at 185 °C for 12 h with air, and OSI was decreased, which is in agreement with results from previous studies [14, 30, 31]. The TBARS content of oxidized maize oil produced in our study was 2.5 times greater than the value reported by Kerr et al. [28] and Hanson et al. [14] using similar thermal processing conditions. The AnV of oxidized maize oil in our study was similar to that reported by Hanson et al. [14], but lower than the value reported by Kerr et al. [28]. Wang et al. reported that the kinetic profile of TBARS reached a plateau of 10.75 mg MDA/kg oil after heating oil at 185 °C for 2 h [32], but our TBARS value was about 10-fold greater than this value after heating at 185 °C for 12 h. In contrast, the oxidized maize oil evaluated in our study contained a lower PV than that reported in other studies [15, 33], while the OSI of oxidized maize oil was greater than reported by others [14, 28, 34]. These results show the difficulty of characterizing the extent of oxidation of lipids because high TBARS infers extensive production of secondary aldehydes from oxidation, but the lower PV and greater OSI values of oxidized maize oil in our study relative to other studies suggests that less oxidation occurred than reported in other studies. Because there were no effects of feeding oxidized maize oil on growth performance of pigs in our study, the PV, AnV, TBARS, and OSI values obtained for oxidized maize oil were not great enough to cause negative performance effects over a 34-day feeding period for nursery pigs.

Synthetic antioxidants are commonly used to increase the oxidative stability of lipids in human foods and feed ingredients. Merrill et al. reported that addition of TBHQ, alone or in combination with other antioxidants, was effective in increasing the stability of high-oleic vegetable oils [35]. We observed that the addition of TBHQ after heating to oxidized maize oil resulted in a lower PV value and greater OSI than maize oil without TBHQ supplementation, which confirms that TBHQ is effective in stabilizing maize oil and increases its resistance to oxidation. Belitz et al. explained that antioxidants delay the rate of oxidation, but do not reverse oxidation if it has occurred [7]. Because TBHQ was added to maize oil after the heating process in the current study, and oil used in phase 2 diets had longer storage time, the magnitude of oxidation of maize oil was greater when added to phase 2 diets than the oil used in phase 1 diets. Therefore, adding TBHQ to oxidized maize oil reduced the magnitude of further lipid oxidation but did not completely prevent it.

Animal physiological status, types of lipids fed, level of oxidation, and multiple combinations of synthetic or natural antioxidants result in variable growth performance responses of nursery pigs fed oxidized lipids. Results from a recent meta-analysis study showed that reductions in growth performance responses from feeding oxidized lipids to pigs and broilers were highly variable, but average reductions in ADG, ADFI, and G:F were 5%, 3%, and 2% respectively, compared with feeding unoxidized lipids [8]. Based on the extent of maize oil oxidation and the heating process used in our experiment, we expected to observe differences in growth performance. However, Chang and van Heugten reported that ADG, ADFI, and G:F were not affected by feeding oxidized maize oil with PV equal to 8.8 mEq O_2_/ kg diet [33]. Similarly, no differences in ADG and ADFI were observed in pigs fed highly oxidized maize oil with PV of 8.1 mEq O_2_/ kg in the diet [15]. In previous work, G:F declined linearly with increasing lipid oxidation [15], but results from the current study showed no dietary effects on G:F. The oxidized maize oil fed in our study had a PV less than 0.4 mEq O_2_ /kg diet and OSI greater than 4.5 h, which may be considered to be mildly oxidized compared with lipids fed in previous studies. However, PV is not a definitive, comprehensive indicator of the extent of oxidation of lipids. The lack of differences in growth performance responses from feeding oxidized maize oil in the current study suggests that the PV, TBARS, AnV, and OSI values of oil may be considered acceptable when evaluating lipid oxidation of maize oil sources. Unfortunately, the health status of the pigs in our study was suboptimal, and acute post-weaning diarrhea resulted in growth depression. As a result, the suboptimal health status of pigs may have compromised our ability to detect differences in growth performance responses from feeding oxidized and unoxidized diets. The overall ADG of pigs fed the unoxidized maize oil in the current study was 360 g/d, which was 5% less than the ADG responses reported by Hanson et al. [14], using the same research facilities and thermal processing conditions to produce oxidized maize oil.

Pigs fed oxidized maize oil in our study had similar feed intake compared with pigs fed oxidized maize oil in our previous study [14]. Several studies have shown that rancid flavor from oxidized lipids [7, 36], aldehyde odor [37, 38], and reduced palatability of oxidation products [39] are possible reasons for reduced feed intake when feeding diets containing oxidized lipids. Furthermore, Dibner et al. reported that lipid oxidation reduced gross energy content by 35% [40], and oxidized vegetable oil had lower nutrient and energy digestibility in poultry [9, 10], suggesting that oxidation reduces the nutritional value of lipids. Our results indicate that the presence of lipid oxidation products in the maize oil fed in the current study were not great enough to cause a reduction in feed intake.

Results from our study showed that adding TBHQ to maize oil was effective in minimizing lipid oxidation by reducing PV up to 37% and OSI up to 69% compared to not using TBHQ in the stored maize oil. However, it is not clear whether TBHQ supplementation to unoxidized and oxidized maize oil has beneficial effects on pigs. Although adding TBHQ to maize oil reduced further lipid oxidation, it had no benefit on growth performance. Chang and van Heugten reported no differences in nursery pig growth performance when an antioxidant blend of ethoxyquin, BHT, and BHA was added at 60 mg/kg to an oxidized maize oil diet [33]. Likewise, McGill et al. observed no differences in ADFI and ADG when broilers consumed oxidized lipid diets supplemented with ethoxyquin at 150 mg/kg [41]. In contrast, adding an antioxidant blend of ethoxyquin and propyl gallate at 135 mg/kg to oxidized soybean oil diets resulted in mitigation of growth rate and feed efficiency reductions in pigs, which was comparable to those fed a maize-soybean meal diet without antioxidants [42], as well as improvements in feed intake and growth rate in broilers [12]. Further studies are needed to evaluate differences in growth performance responses of pigs when adding various types of synthetic antioxidants to oxidized lipid diets. However, it is important to recognize that the U.S. Food and Drug Administration strictly regulates the use of synthetic antioxidants in foods and animal feeds. The maximum usage rate of TBHQ is to not exceed 0.02% of the lipid content of food or feed. The maximum inclusion level for ethoxyquin is 150 mg/kg, and 200 mg/kg for BHA and BHT [43]. These restrictions are based on safety concerns from excessive use because metabolites of antioxidants can be cytotoxic [44] and can result in DNA damage [20]. In addition, oxidative stress can be triggered by inappropriate antioxidant usage [45] and an overdose of antioxidants can lead to an oxidant-antioxidant imbalance [43, 46]. Therefore, prudent use of synthetic antioxidants in animal feeds is warranted to avoid adverse physiological effects from excessive use.

The trend for increases in HSI observed in pigs fed oxidized maize oil in our study is consistent with previous findings [47, 48]. An increase in liver size relative to body weight has been used as an indicator of toxicity [49], and studies have shown that feeding oxidized lipid diets increases HSI [50]. This response may be a result of increased synthesis of microsomal enzymes to mitigate toxicity [47]. Toxic oxidized products can be absorbed and transported to liver [51], and broilers that consumed oxidized lipids have been shown to have increased hepatocyte proliferation [52]. Increased hepatocyte proliferation may be related to cytotoxicity of oxidized products because elevated plasma alanine transaminase has been observed in pigs fed oxidized soybean oil [42]. Studies have also shown that liver weight was positively correlated with active oxygen method, AnV, and TBARS [48], and negatively correlated with growth rate in swine [42].

The reduction in serum vitamin E concentrations from feeding oxidized maize oil in our study is consistent with reports from several other studies [10, 14, 16, 33, 53], but it may be inappropriate to compare changes in vitamin E concentrations among studies due to varying levels of oil oxidation used. However, our results are in agreement with results reported by Hanson et al. [14], which used similar experimental conditions and found serum vitamin E concentration to be 0.3 μg/mL in nursery pigs fed diets containing 6% oxidized maize oil. The physiological antioxidant defense system requires involvement of vitamin E (non-enzymatic antioxidant) and Se (structural component of glutathione peroxidase) [28]. On average, pigs fed oxidized lipids have a 46% reduction in serum vitamin E content compared with pigs fed unoxidized lipids [8]. Our findings indicate that even with moderately low oxidation of the maize oil fed to weaned pigs, the reduction in serum vitamin E concentrations were not recovered by adding TBHQ to maize oil. There are 3 possible explanations for this reduction in serum vitamin E. First, high metabolic demands reduce serum vitamin E concentration because it interacts with free radicals produced from oxidation to minimize the negative effects of endogenous oxidative stress [16]. Second, thermally oxidized lipids decrease the concentration of tocopherols [30, 53]. Lastly, thermally oxidized lipids have decreased α-tocopherol digestibility [54], resulting in animals absorbing less vitamin E. Unlike vitamin E, there were no oxidation and antioxidant differences in serum and liver Se concentrations. Hanson et al. reported no effect of increasing dietary levels of oxidized maize oil on liver Se concentration, but there was a trend for reduced Se concentration in serum [14]. Similar to our results, Tabatabaei et al. found no differences in serum and liver concentration of Se in rats fed either fresh or oxidized sunflower oil [55]. Therefore, serum vitamin E concentration appears to be a more sensitive and appropriate marker than Se for evaluating oxidative status of pigs fed oxidized maize oil.

## Conclusions

Heating maize oil at 185 °C with 12 L/min of continuous air for 12 h achieved moderate oxidation, and the addition of TBHQ was partially effective in preventing further oxidation as measured by oil stability index of unoxidized and oxidized maize oil. The PV, TBARS, and AnV values of the oxidized maize oil fed in this study may serve as a general guide for estimating threshold levels of oxidation products that do not reduce growth performance of weaned pigs. However, the long-term effects of feeding this source of oxidized maize oil are unknown because diets were only fed for 34 days, but the trend for greater HSI and reduced serum vitamin E concentration in pigs fed oxidized maize oil suggests potential negative health and performance effects. These effects were not prevented by the addition of TBHQ to oxidized maize oil. The addition of TBHQ to maize oil partially protected oils against further oxidation but did not affect growth performance of nursery pigs.

## Abbreviations

ADFI: Average daily feed intake
ADG: Average daily gain
AnV: p-Anisidine value
AOCS: American Oil Chemists’ Society
BHA: Butylated hydroxyanisole
BHT: Butylated hydroxytoluene
BW: Body weight
FDA: Food and Drug Administration
G:F: Gain to feed ratio
HIS: Hepatosomatic index
HPLC: High-performance liquid chromatography
MDA: Malonaldehyde
OSI: Oil stability index
PV: Peroxide value
Se: Selenium
TBARS: Thiobarbituric acid reactive substances
TBHQ: Tert-butylhydroquinone

## Authors’ information

Yuan-Tai Hung, is a Ph.D. candidate at the University of Minnesota with interest in swine nutrition and intestinal physiology.

Andrea R. Hanson, Ph.D., is a swine nutritionist at Professional Swine Management, LLC in Carthage, IL with expertise in nutrient digestion and utilization, and lipid oxidation and utilization.

Pedro E. Urriola, Ph.D., is a Research Assistant Professor of Animal Science at the University of Minnesota with expertise in nutrition, nutrient digestion and utilization, and dietary fiber in alternative feed ingredients for pigs.

Lee J. Johnston, Ph.D., is a Professor of Animal Science at the University of Minnesota with expertise in gilt development, sow nutrition, and nutrition/environment interactions.

Brian J. Kerr, Ph.D., is a lead scientist at the USDA-Agricultural Research Service since 2001 with expertise in non-ruminant nutrition research.

Gerald C. Shurson, Ph.D., is a Professor of Animal Science at the University of Minnesota with expertise in nutrition, energy and nutrient digestibility, nutrient utilization, lipid oxidation and utilization, and alternative feed ingredients in swine diets.
